# Honey bee (*Apis mellifera*) queen quality: host-microbial transcriptomes exploring the influence of age and hindgut symbiont *Commensalibacter melissae*

**DOI:** 10.1186/s42523-025-00408-w

**Published:** 2025-05-02

**Authors:** Duan C. Copeland, Oliver L. Kortenkamp, Brendon M. Mott, Charles J. Mason, Kirk E. Anderson

**Affiliations:** 1 USDA-ARS Carl Hayden Bee Research Center, 2000 E. Allen Rd, Tucson, AZ 85719 USA; 2 Department of Entomology and Center for Insect Science, University of Arizona, Tucson, AZ 85721 USA; 3 USDA-ARS Daniel K. Inouye U.S. Pacific Basin Agricultural Research Center, 64 Nowelo St., Hilo, HI 96720 USA

**Keywords:** Honey bee, Queen quality, *Commensalibacter*, RNA-seq, Keyword_5_

## Abstract

Understanding the biological mechanisms underlying extreme lifespan variation within species remains a fundamental challenge in aging research. Here, we investigated the role of gut microbiota and age in honey bee (*Apis mellifera*) queens combining 16S rRNA gene sequencing and transcriptomics. Analysis of 40 queen hindguts revealed that *Commensalibacter melissae* (Alpha 2.1) relative abundance was significantly higher in young queens compared to old queens. Using queens with the highest and lowest *C. melissae* relative abundance, RNA sequencing identified 1451 differentially expressed genes associated with *C. melissae* abundance, twice the number associated with age alone (719 genes). Queens with high *C. melissae* abundance showed distinct transcriptional profiles related to stress response, protein homeostasis, and longevity-regulating pathways, particularly genes involved in oxidative stress response and cellular maintenance. Our analysis revealed complex relationships between age, *C. melissae* abundance, and gene expression patterns, suggesting that multiple interacting factors contribute to queen quality. These findings contribute to our understanding of host-microbe interactions in honey bee queens and highlight the intricate relationship between gut microbiota composition and host physiology in honey bees.

**Supplementary Information:**

The online version contains supplementary material available at 10.1186/s42523-025-00408-w.

## Introduction

Honey bees (*Apis mellifera*) are essential pollinators in both natural ecosystems and agricultural settings, playing a crucial role in global food security and biodiversity. At the heart of every honey bee colony is the queen, whose extraordinary longevity and reproductive output are key to colony success [61]. In the somewhat recent past, queens were renowned for their exceptional longevity and in extreme cases living up to 8 years compared to the typical 4–6 weeks lifespan of workers [54]. This striking difference in lifespan between queens and workers has long intrigued researchers, and while queen quality is known to be affected by various factors, including genetics [36, 56], matedness [18, 67], and environmental conditions [50], the gut microbiome is a relatively understudied factor that may contribute to queen quality and longevity, with some recent work beginning to explore this area [3, 15, 20, 22]. A survey of American beekeepers [69] identified queen-related issues as a major problem for the sustainability of commercial beekeeping. In response, commercial operations have adopted systematic queen replacement practices, often sourcing queens from Hawai’i when they are seasonally unavailable from California. This shift to year-round queen breeding has been supported by increased pollination fees, facilitating an industry-wide strategy of annual queen replacement. In a large longitudinal study of queen quality [58], we found that first-year queens outperformed second-year queens, validating beekeepers'queen replacement practices. Furthermore, we discovered that the gut microbiome of recently mated queens contained higher levels of Alpha 2.1 *Commensalibacter*, an acetic acid bacteria that depletes rapidly with age [3].

The gut microbiome is increasingly recognized for its role in modulating host physiology, metabolism, and immune function in honey bees [2, 9, 31]. The queen's gut microbiome differs significantly from that of worker bees, with queens harboring a less diverse but highly predictable and specialized bacterial community [3, 20, 42, 66]. Among the simple microbial communities inhabiting the queen gut, *Commensalibacter melissae* (previously known as Alpha 2.1 [11, 24, 52]) has emerged as a key symbiont with potential implications for queen health and longevity. As an acetic acid bacterium, *C. melissae* typically thrives in carbohydrate-rich, acidic environments [25], that may change as queens age. Previous studies have demonstrated that the relative and absolute abundance of *C. melissae* decreases significantly throughout the alimentary tract as queens age [3]. It has also been shown that *C. melissae* decreases rapidly in abundance when queens are placed into a queen bank, an environment of low metabolic demand where queens are fed by workers, but do not lay eggs [20].These results suggest a possible link between *C. melissae* abundance and queen metabolism associated with aging.

In this study, we define queen quality as encompassing several measurable aspects including mating success, reproductive output, longevity, colony performance, and molecular indicators of cellular health. While queen quality includes multiple factors and can be difficult to quantify [1], we specifically focus on age-related aspects of quality as indicated by oxidative stress markers and gene expression profiles. Similarly, perhaps the queen’s distinct microbiome, coupled with the unique diet of royal jelly, contributes to her quality and extended lifespan through enhanced metabolic efficiency and improved immune function [3]. Understanding these microbiome-mediated mechanisms could not only illuminate the evolutionary basis of caste-specific longevity but also provide valuable insights for honey bee health management and conservation efforts. Currently, queen failures consistently rank as one of the most prominent causes of yearly colony losses across the US [5, 13]. To combat these losses, beekeepers often replace queens annually, as younger queens (containing a greater abundance of *C. melissae*) generally outperform older queens in terms of egg production [58].

Transcriptional analyses have proven valuable for understanding honey bee biology, revealing how nutrition affects worker development [23] and identifying key gene expression differences between queens and workers [16, 32]. These studies demonstrate the utility of transcriptomics for understanding age-related and developmental processes in honey bees. In this study, we combined 16S rRNA gene sequencing and transcriptomics to explore the role that *C. melissae* has in modulating host queen physiology with respect to age. We use protein carbonyl accumulation in the fat body as a biomarker of biological aging, complemented by transcriptional signatures that reflect physiological status independent of chronological age. We collaborated with a commercial beekeeping operation in Illinois to select a cohort of 40 mixed aged queens. We analyzed *C. melissae* abundance and gene expression profiles of first year versus second year queens. We explored differences in gene expression patterns and fat body carbonyl accumulation to provide insights into interactions between the gut microbiome and age of honey bee queens. We show that *C. melissae* abundance is associated with distinct transcriptional profiles related to stress response and cellular maintenance, suggesting this symbiont may play a key role in queen health and longevity. Understanding these interactions could pave the way for novel strategies to enhance queen longevity and improve overall colony performance.

## Methods

### Queen sampling

Queens were sourced from two locations: the USDA-ARS Carl Hayden Bee Research Center in Arizona (n = 13) and a commercial beekeeping operation in Illinois (n = 27). Arizona queens were all established and had survived more than 1 year in their colonies. Illinois queens represented a mixed-age population, consisting of both newly introduced queens (requeened in spring 2023, April–May) and older queens that had not been requeened that spring, though natural supersedure could not be ruled out. All queens were sampled in June 2023 from robust double-deep colonies. The selected colonies were highly productive with strong populations, often reaching space limitations for both brood production and honey storage. To ensure sample quality and consistency, we specifically selected queens from thriving colonies and deliberately excluded any colonies showing signs of queen failure or irregular egg-laying patterns. Forty queens in total were collected into sterile 2.0-ml tubes and immediately frozen on dry ice and stored at − 80 °C for nucleic acid extraction.

### Dissections and tissue collection

Queens were pinned through the thorax in 70% ethanol to wash and aid in dissection. Micro-dissection scissors were used to cut through the sides of the abdomen to access the digestive tract. The entire digestive tract was removed and floated in ethanol to manually separate the gut tissues with dissection tweezers. *C. melissae* is most abundant in the hindgut, so we targeted the ileum and rectum together for analysis. The abdominal fat body was extracted as a single unit for use in gene expression and protein oxidation assays to assess biological aging.

### Nucleic acid extractions

Queen fat body, ileum, and rectum tissues were each bead-beaten separately in 1X TE buffer for 2 min at 30-s intervals and centrifuged at 30 s at 3000 rcf to recover the supernatant. To extract nucleic acids from the gut tissues (DNA and RNA simultaneously), we used Qiagen AllPrep PowerViral DNA/RNA Kit (Qiagen, Hilden, Germany) following the manufacturer's protocol and methodology also reported in [21]. 20 µL of ileum and 20 µL rectum elution from the extractions were then pooled into hindgut samples for downstream sequencing and analysis.

### Carbonyl protein oxidation assay for biological age assessment

The fat body supernatant fraction was used in a protein oxidation assay to quantify the accumulation of protein carbonyl groups associated with oxidative stress and aging [57], as in [21]. We used this method as a biomarker of biological aging based on the established relationship between oxidative stress and the aging processes [34, 37], measuring protein carbonyl accumulation in fat body tissue as a validated biomarker of aging in honey bees, as established in our previous work [3, 21]. This method provides an objective measure of biological aging that correlates with chronological age but also accounts for individual variation in aging rates. For queens from the Illinois commercial operation, we used the operator's records of queen introduction dates to supplement our carbonyl data, categorizing queens as'young'(introduced in spring 2023, with lower carbonyl levels) or'old'(introduced before spring 2023, with higher carbonyl levels). The carbonyl assay was particularly valuable for identifying cases where biological age did not align with chronological records, potentially due to natural supersedure events that occurred without beekeeper intervention. Protein oxidation was expressed as nanomoles of carbonyl groups per mg of protein.

### DNA sequencing and 16S rRNA gene community analysis

DNA from gut tissues was amplified in a single step procedure to amplify full-length 16S rRNA (V1-V9) using degenerate primers 27 F (GCATC/barcode/AGRGTTYGATYMTGGCTCAG) and 1492R (GCATC/barcode/RGYTACCTTGTTACGACTT). PCR was performed with Q5 2 × Hot Start High-Fidelity Master Mix (New England Biolabs) using the following conditions: 98 °C 30 s; 98 °C 10 s, 55 °C 30 s, and 72 °C 2 min for 22 cycles; final extension of 72 °C for 10 min. Reactions (30 µL) were performed following manufacturer recommended master mix concentrations, with primer concentrations of 250 nM. Positive (ZymoBiomics Microbial Community DNA Standard; Zymo Research) and negative, non-template controls were included as process controls. After PCR amplification, target amplicons were purified from residual primers and primer-dimer using an AMPure bead cleanup and DNA concentrations were determined using a Qubit fluorometer (Thermo Fisher). Samples were then pooled (~ 3 ng per sample) and were prepared for sequencing by generating a SMRTbell library with a Pacific Biosciences SMRTbell prep kit 2.0 using manufacturer suggested inputs and procedures. Amplicons were sequenced on a single Pacific Biosciences 8M SMRT Cell on a PacBio Sequel IIe (Pacific Biosciences) at USDA-ARS PBARC (Hilo, HI). After sequencing, circular consensus sequences from the subreads were obtained using the SMRTLink v8.0 software.

Full length 16S rRNA gene sequence data were processed using MOTHUR v.1.43 [60] according to previously published protocols [21]. Briefly, sequence barcodes (Table S1) were removed using the ‘fastq.info’ command. Next ‘screen.seqs’ was used to remove ambiguous bases with maxambig = 0. Unique sequences were generated using the ‘unique.seqs’ command, followed by the generation of a count file containing group information using the ‘count.seqs’ command. Sequences were aligned to the full-length 16S rRNA BEExact database [27] using the ‘align.seqs’ command. Sequences were filtered to remove overhangs at both ends and gaps using ‘filter.seqs’. The unique.seqs command was repeated to remove new redundancies from filtering. A precluster step using ‘pre.cluster’ was performed before using the ‘chimera.vsearch’ command [59] to identify chimeric sequences. The command ‘remove.seqs’ was used to remove the identified chimeric sequences. Next, sequences were classified at the unique level with the BEExact database using ‘classify.seqs’ command. Sequences not of bacterial origin (fungi, archaea, mitochondria, and chloroplasts) were removed using the ‘remove.lineage’ command. Unique sequences within our count table that were single or doubletons (having only one or two members) were removed using the AWK command in UNIX. Next, we utilized the ‘list.seqs’ and ‘get.seqs’ commands to generate inputs for our distance matrix. The distance matrix was constructed for the aligned sequences using the ‘dist.seqs’ command. Following this, we employed the ‘cluster’ command to group the sequences into unique operational taxonomic units (OTUs), generated a shared file with the ‘make.shared’ command, and assigned taxonomic classifications to each OTU using the ‘classify.otu’ command. Unique sequences were consolidated at the species level using the ‘merge.otus’ command.

The 16 most abundant operational taxonomic units (OTUs) and a sum of remaining OTUs were normalized by qPCR BactQuant [47] absolute abundances by first calculating the proportion of each OTU by dividing the raw read count into the total number of sequences per sample. Each ratio was multiplied by the total BactQuant 16S rRNA gene copies qPCR for each sample. Next, each OTU was corrected for 16S gene copies per bacterial cell; 16S rRNA gene copy number were assigned based on the exact match or closest taxonomic representative [65]. The summed column of remaining OTUs were assigned 4.2 gene copies, the mean value of 16S rRNA gene copy number in bacteria [70]. Next, the data were CLR-transformed using the software CoDaPack [19].

### Total bacterial quantification

Using purified total DNA, we quantified total bacterial abundance for the hindgut using a quantitative PCR (qPCR) assay of the 16 rRNA gene [47]. We created a standard curve using a tenfold serial dilution series of a plasmid standard containing a full-length Escherichia coli 16S rRNA gene sequence. We amplified a 466 bp fragment in the V3–V4 region of the 16S rRNA gene using universal primer pair (5′-CCTACGGGDGGCWGCA-3′ and 5′-GGACTA CHVGGGTMTCTAATC-3′). PCR reactions were performed in triplicate on a BioRad CFX96 (Biorad, Hercules, California, US) as follows: 12 μl reactions containing 9 μl of iTaq Universal SYBR Green Supermix (BioRad, Hercules, California, US), 0.5 μl forward primer, 0.5 μl reverse primer, and 2 μl of DNA template. The cycling conditions were 95 °C for 3 min followed by 40 cycles of 95 °C for 10 s and 60 °C for 60 s. The qPCR results were expressed as the total number of 16S rRNA gene copies per DNA extraction (200 μl volume elution).

### RNA sequencing and transcriptomic analysis

RNA from extracted samples was processed for sequencing by depleting the rRNA from samples using a RiboFree cDNA Kit (Zymo Research). Samples were pooled in equimolar concentrations and libraries were prepared for sequencing using an Adept Rapid PCR-Plus Kit (Element Biosciences). Libraries were sequenced on an Element Biosciences AVITI sequencer using an AVITI 2 × 150 Cloudbreak High Output sequencing kit at USDA-ARS PBARC. One queen sample dropped out of sequencing due to insufficient cDNA yield, leading to a failure in library preparation. Consequently, this sample was excluded from downstream RNA-seq analyses.

All paired-end raw reads were filtered and trimmed using Trimmomatic v0.38 [8] with the following parameters: LEADING:3 TRAILING:3 SLIDINGWINDOW:4:15 MINLEN:75. FASTQC was used to ensure quality control [6]. Kraken2 was used to map and split reads to the *Apis mellifera* genome assembly Amel_HAv3.1 (PRJNA471592) and the gut symbiont *Commensalibacter melissae* (PRJNA495947) [72]. *A. mellifera* reads were mapped against *A. mellifera* genome using STAR v2.7.10b [30] and *C. melissae* reads were mapped to *C. melissae* genome using Bowtie 2 v2.5.2 [44]. Gene counts were obtained using Subread v2.0.4 package FeatureCounts [45]. Genes with read counts below 4 were removed, and genes with variance less than 15% across samples were filtered out. Final counts were normalized by employing a log2-counts per million (logCPM) transformation.

### Quantitative real-time PCR (qRT-PCR)

To validate differentially expressed genes between ‘low’ and ‘high’ *Commensalibacter* groups we performed qPCR on a select group of genes. A cDNA template was generated from the purified RNA fraction. Briefly, RNA was converted into cDNA with Thermo Scientific RevertAid First Strand cDNA Synthesis Kit (Thermo Fisher Scientific, Waltham, Massachusetts, United States) following the manufacturer’s instructions. PCR reactions were performed using gene-specific primers and the following protocol: initial denaturation at 95 °C for 5 min; 40 cycles with denaturation at 95 °C for 15 s; and a combined annealing and extension step at 58 °C for 30 s. The reactions were carried out using iTaqTM Universal SYBR® Green Supermix (Biorad, Hercules, California, US) in triplicate on an CFX96 TM Real-Time PCR Detection System (Biorad, Hercules, California, US). To confirm the absence of contaminant DNA and primer dimers, we used no-template controls made of water and analyzed melt-curves for each qPCR plate. Relative gene expression was calculated using the 2^–∆∆Ct^ method [48] using both β-actin and RPS18 as reference genes [41]. Then the data were normalized with a log10 transformation for downstream analyses. A list of primers used in this study is available in Table S1. Primers not sourced from previous studies were designed with NCBI Primer-BLAST software (https://www.ncbi.nlm.nih.gov/tools/primer-blast/). Gene targets were selected from lists of differentially expressed genes between ‘low’ and ‘high’ *Commensalibacter melissae* groups for *C. melissae* and *Apis mellifera* genes.

### Statistical analysis

A multivariate analysis of variance (MANOVA) was performed on CLR-transformed data with OTUs 1 to 17 as dependent variables using SAS_v9.4 [40]. Using carbonyl to approximate age, we binned the queens into young and old categories, examining age as an independent variable. Normalized absolute abundance data were also analyzed by age using ANOVA. Normalized qRT-PCR results were analyzed using linear regression models in JMP v14.3.0 (JMP_1989–2007), with gene expression as the dependent variable and *C. melissae* relative abundance as the independent continuous variable. This approach allowed us to examine the continuous relationship between gene expression and symbiont abundance without relying on categorical binning. *P* values are reported for the significance of the regression slope. A false discovery rate (FDR) was employed to account for multiple comparisons. We also conducted Pearson correlation analysis to examine the relationship between carbonyl levels and *C. melissae* relative abundance using JMP v14.3.0. The correlation coefficient (r) and associated *p* values were calculated to determine the strength and statistical significance of the relationship.

Differential gene expression analysis was performed with the Bioconductor package DESeq2 [49] in ExpressAnalyst [33]. We used source (AZ and IL) as a blocking variable for all downstream analysis. While filtered counts were used for differential expression analysis with DESeq2, normalized data was used only for downstream data visualizations. Differentially expressed genes (DEGs) were calculated based on contrasting groups; young versus old host age, or low versus high *Commensalibacter* relative abundance. Initially, carbonyl expression was used to bin queens into young and old and DEGs were calculated. Then, to emphasize the contrasting groups, the original dataset was reduced to 24: the 12 youngest and 12 oldest queens. Likewise, to quantify DEGs among queens with varied *Commensalibacter* relative abundance, 24 were selected based on the following criteria: nine queens with high abundance >60% were compared to 15 queens with low <30% relative abundance. DEGs of *Commensalibacter melissae* were assessed within the same cohort of low and high *Commensalibacter* queens. To reduce the chance for type 1 errors in multiple hypothesis testing, we employed Benjamini–Hochberg method to correct for FDR. DEGs were considered significant if the adjusted *p* value was < 0.05.

The list of DEGs were uploaded into the Database for Annotation, Visualization and Integrated Discovery (DAVID) v2024q1 [39, 62] for functional annotation clustering analysis. We used the *Apis mellifera* genome (12,313 genes recognized by DAVID) as the background gene list against which enrichment was tested, with only 38 genes from our dataset not recognized by the database. DAVID employs a modified Fisher’s exact test (EASE score) to determine enrichment, comparing the proportion of genes in our list associated with a particular Gene Ontology (GO) term to the proportion in the background genome. We applied the Benjamini–Hochberg procedure to control the false discovery rate, with an adjusted *p* value threshold of 0.05 for significance. The analysis grouped genes into GO terms, Kyoto Encyclopedia of Genes and Genomes (KEGG) pathways, and UniProtKB (UniProt Knowledgebase) (UP) keywords (KW).

We used a heatmap to visualize DEGs with a hierarchical clustering analysis grouped with Ward’s clustering method, which groups queens and genes based on their similarity rather than their associated metadata label (age or *Commensalibacter* relative abundance) [29]. Normalized RNA-seq counts were sub-selected from significant DEGs to create principal component analyses (PCA) based on our results from Ward’s clustering analysis.

## Results

### Microbial community analysis

**Fig. 1:**
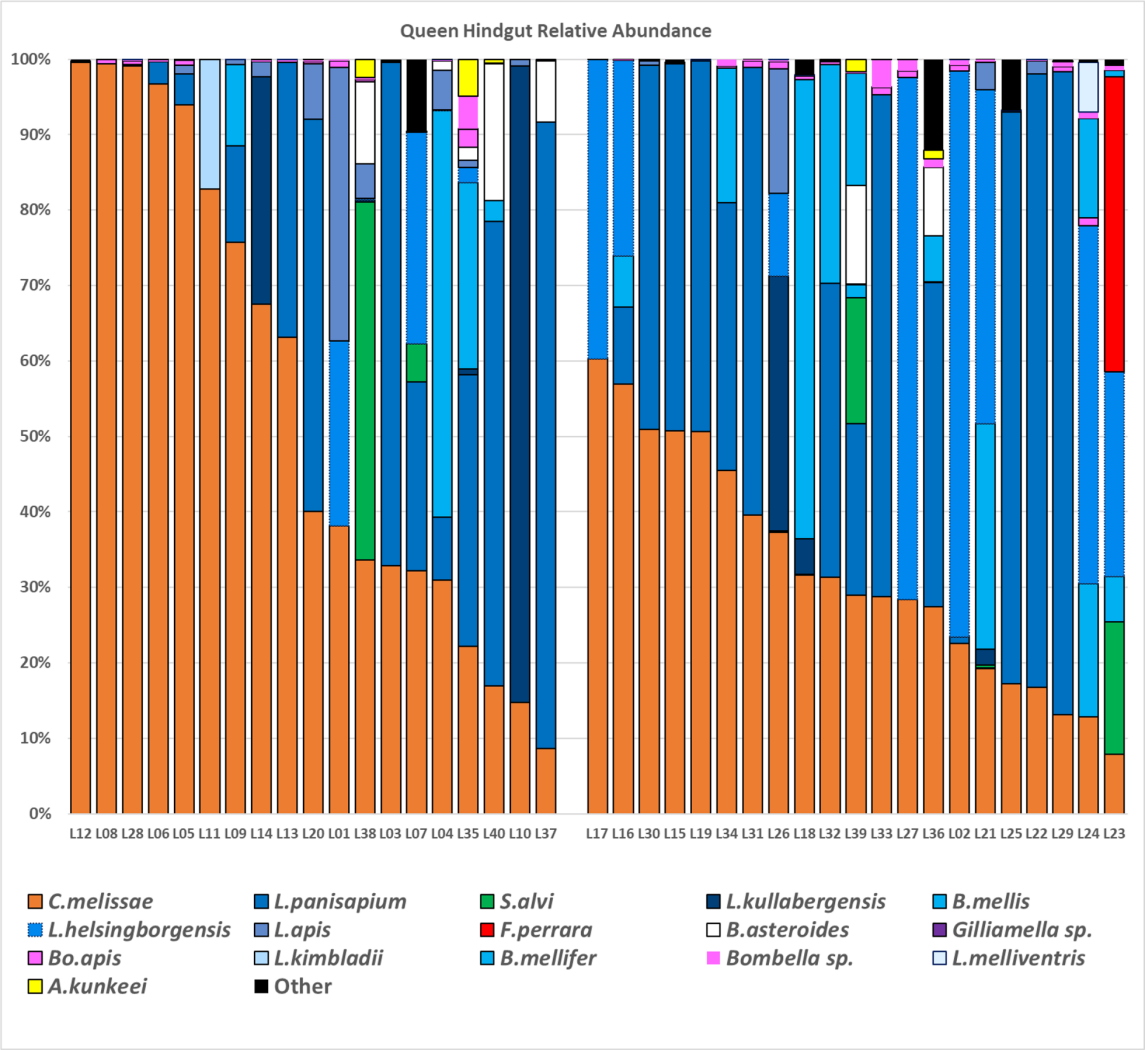
Relative abundances of queen hindgut microbiotas. Color-coded bars represent relative abundance corrected by species-specific 16S rRNA gene copy number. All 40 queen samples binned within each age group (young and old) are arranged in descending *C. melissae* relative abundance to highlight the difference in *C. melissae* prevalence between young and old queens. The ‘Other’ category represents the sum of all bacterial taxa that contributed less than 0.5% to the total community abundance

PacBio sequencing returned 816,143 full-length 16S rRNA genes across 40 libraries from the hindguts of queens (Table S2). The queens were binned into young (n = 20) and old (n = 20) groups based on carbonyl protein oxidation levels in fat body tissue, which served as a biomarker of biological aging. Bacteria were classified using the BEExact database to match the species level [27]. Libraries averaged 19.3 K reads and the number of OTUs went from 439 to 62 after OTUs were merged at the species level. After quality review and removal of contaminants, 16 OTUs were retained which accounted for 99.5% of all reads, while a 17 th OTU comprised of the remaining OTUs included the last 0.5%. The BEExact database, initially identified our primary symbiont as *Commensalibacter* unknown. In a separate analysis, we performed a BLAST analysis of the full-length 16S rRNA gene sequence against the NCBI database, which revealed > 99% sequence identity to *Commensalibacter melissae*. Following the recent taxonomic designation by Botero and Vandamme [11], we use *C. melissae* throughout this manuscript when referring to the previously known Alpha 2.1 symbiont. Approximately 73% of reads belonged to the gut symbionts *C. melissae* and *Lactobacillus panisapium* (35.4% and 37.7%, respectively) (Fig. 1). A one-way MANOVA was performed on CLR-transformed data with OTUs 1 to 17 as dependent variables. The MANOVA examined age as an independent variable (young vs old) and as a continuous variable with carbonyl groups per mg of protein. Overall, the model was not significant for age (F ratio = 0.55, Pr > F = 0.1355). When considering only *C. melissae*, we found that its relative abundance was significantly different between young and old queens (F ratio = 4.7745 Prob > F = 0.0351; Figure S1) but not the absolute abundance (F ratio = 2.1849 Prob > F = 0.1476; Figure S1). Pearson correlation analysis was conducted to examine the relationship between carbonyl levels and *C. melissae* relative abundance across all queens. We found that the correlation was weak and not statistically significant (r = − 0.2497, *p* = 0.1203), suggesting that while categorical comparisons show significant differences in *C. melissae* abundance between young and old queens, the relationship is more complex than a simple linear correlation when examined as continuous variables.

We compared the total bacterial abundance in the hindgut between young and old queens using qPCR quantification of 16S rRNA gene copies. Young queens had similar total bacterial loads (mean ± SE: 1.09E + 08 ± 1.44E + 07 16S rRNA gene copies) compared to old queens (mean ± SE: 1.34E + 08 ± 2.57E + 07 16S rRNA gene copies; *p* = 0.6650). This finding indicates that while the relative abundance of specific taxa like *C. melissae* changes with age, the overall size of the bacterial population remains stable in the queen hindgut.

### Overview of RNA-seq data and differentially expressed genes analysis

On average, 18.3 million reads were generated from each queen host library across 12,356 genes (Table S3). Reads were also mapped to *Commensalibacter melissae* which averaged 2.3 million reads per library across 1839 genes (Table S4). On average, 75.15% of reads were mapped to the *Apis mellifera* genome [26, 71]. The remaining reads were bacterial (16.76%, 5.48% of which belonged to *C. melissae*), viral (1.27%), and fungal (0.08%) in origin.

**Fig. 2:**
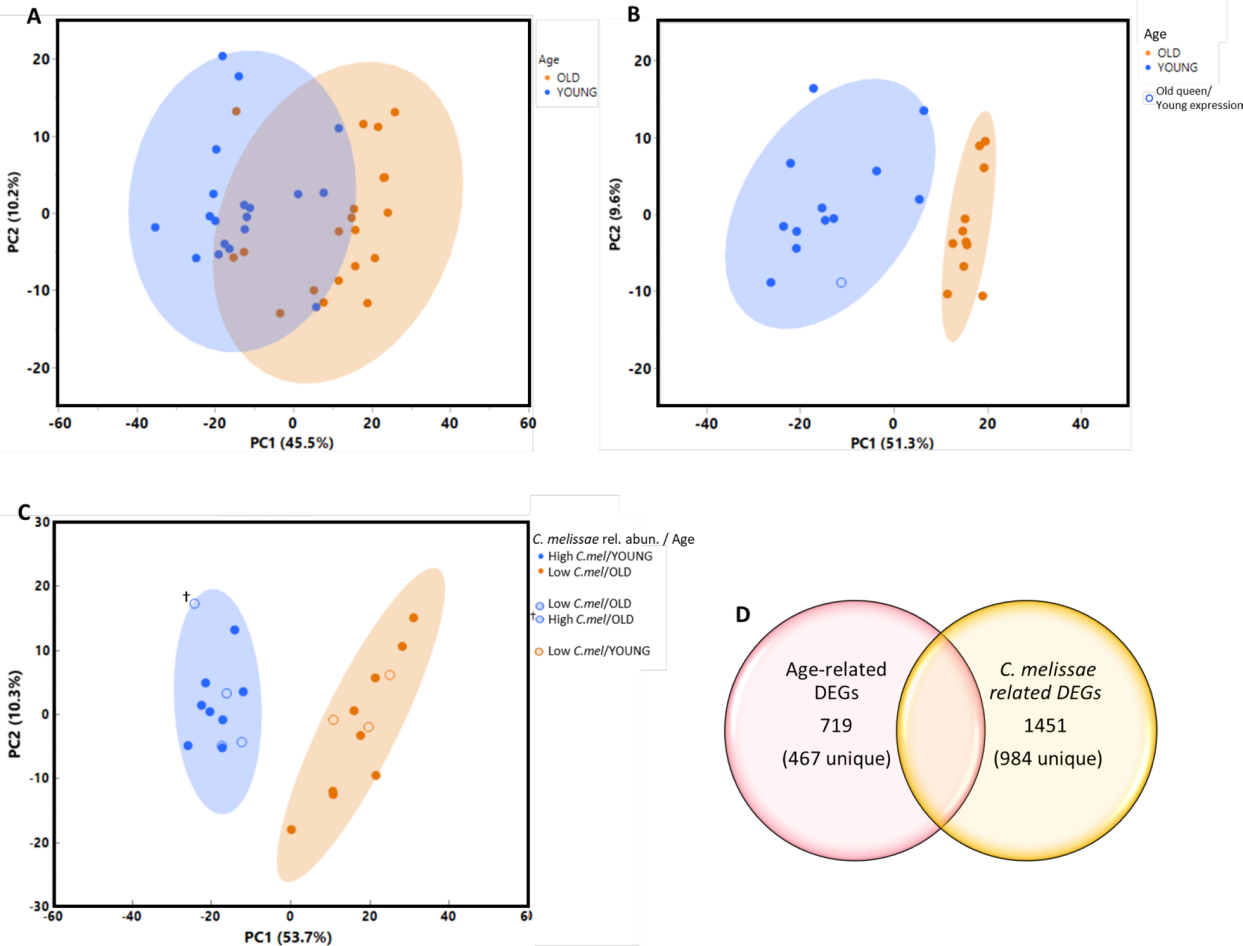
Principal component analysis of differentially expressed genes (DEGs). Clustered groups of points contain similar gene expression. Density ellipses cover 95% of plots for each group. Queens were classified as'young'or'old'based on carbonyl levels in fat body tissue, with a threshold of 15.0 nmol/mg protein separating the groups. **A** 680 DEGs associated with queen age (young vs old) of 39 queens. **B** 24 queens sub-selected for youngest versus oldest queens represent 719 DEGs using Ward’s clustering method. Open circle indicates an outlier queen who was biologically old, but showed gene expression patterns like young queens. **C** 24 queens sub-selected for low (< 30% relative abundance) and high (> 60% relative abundance) of gut symbiont *Commensalibacter melissae* represent 1451 DEGs using Ward’s clustering method. Open circles indicate queens whose expression patterns did not align with expectations: biologically young queens with low *C. melissae* had gene expression patterns like old queens; two old queens had low *C. melissae*, but gene expression like young queens; and one biologically old queen had high *C. melissae*, and gene expression like young queens. **D** Venn diagram showing the relationship between differentially expressed genes (DEGs) identified in our two main comparisons. The left circle represents the 719 DEGs found when comparing young versus old queens based on biological age (carbonyl levels). The right circle represents the 1451 DEGs found when comparing queens with high versus low *C. melissae* abundance. The overlap (252 shared DEGs) indicates genes affected by both age and *C. melissae* abundance, while the non-overlapping portions represent genes uniquely associated with either age (467 DEGs) or *C. melissae* abundance (984 DEGs)

For our RNA-seq analysis, we used two complementary approaches to categorize queens: We initially classified 39 queens as “young” or “old” based on protein carbonyl oxidation of fat body tissue, which serves as a validated biomarker of biological aging. Queens with carbonyl levels below 15.0 nmol/mg protein were classified as biologically"young"(n = 20), while those above this threshold were classified as biologically"old"(n = 19). This approach identified 680 differentially expressed genes (DEGs) (Table S5, P_adj_ < 0.05). Next we performed a refined age comparison. DEGs were inspected by principal component analysis (PCA), which shows a clear separation of the samples based on chronological age (Fig. 2A). To enhance the contrast between distinctly young and old queens, we subsequently selected the 12 queens with the lowest carbonyl levels (< 13.3 nmol/mg protein) as a refined "young" group and the 12 queens with the highest carbonyl levels (> 18.5 nmol/mg protein) as a refined "old" group. As expected from this selection approach, DESeq2 analysis of this subset showed more distinct separation between age groups, with 719 DEGs. (Table S6, P_adj_ < 0.05). For our analysis of age-associated gene expression patterns, we classified these clusters as representing "youthful" and "aged" transcriptional profiles. The list of DEGs were inspected by PCA, revealing tighter clustering of samples that aligned with age (Fig. 2B).

Next, to further investigate the relationship between *C. melissae* relative abundance and queen hindgut gene expression we selected 24 queens based on *C. melissae* relative abundance regardless of age: 9 queens with high abundance (> 60% relative abundance) and 15 queens with low abundance (< 30% relative abundance) (Figure S2). This comparison yielded 1451 DEGs (Table S7, P_adj_ < 0.05). The PCA of *C. melissae* abundance clustered into two distinct ellipses (Fig. 2C). The clusters had unique highlights; queens that were biologically young with low *C. melissae* had aged gene expression patterns like old queens with low *C. melissae*. Two older queens had low *C. melissae*, but still had youthful gene expression like young queens. Finally, there was one old queen with high *C. melissae* relative abundance whose gene expression patterns were youthful and matched young queens with high *C. melissae*. A Venn diagram was constructed to examine the intersection between age-related and *C. melissae* abundance-related gene expression patterns (Fig. 2D) (Table S8). Out of the 719 DEGS associated with age, 467 were expressed uniquely in the young versus old queen comparison. For *C. melissae*, 984 of the 1451 DEGs were expressed uniquely in queens with low versus high *C. melissae* abundance. There were 252 DEGs shared between both analyses.

After examining the relationship between *C.* *melissae* abundance and queen gene expression patterns, we next investigated changes in the bacterium’s own gene expression. By analyzing RNA sequences mapped to the *C. melissae* genome, we identified bacterial genes that were differentially expressed between queens with low and high *C. melissae* abundance. This allowed us to examine how the bacterial transcriptome differs based on relative abundance and potentially identify metabolic functions or pathways in *C. melissae* that might influence queen physiology and contribute to the observed differences in host gene expression. DESeq2 analysis on *C. melissae* mapped genes related to low and high *C. melissae* relative abundance resolved 9 DEGs after FDR correction (Table S9). Four out of nine genes coded for16S ribosomal RNA. The remaining five genes were TonB-dependent receptor, BadF/BadG/BcrA/BcrD ATPase family protein, ribonuclease J, helix-turn-helix transcriptional regulator, and aldo/keto reductase.

### Gene ontology terms and KEGG pathways

**Fig. 3:**
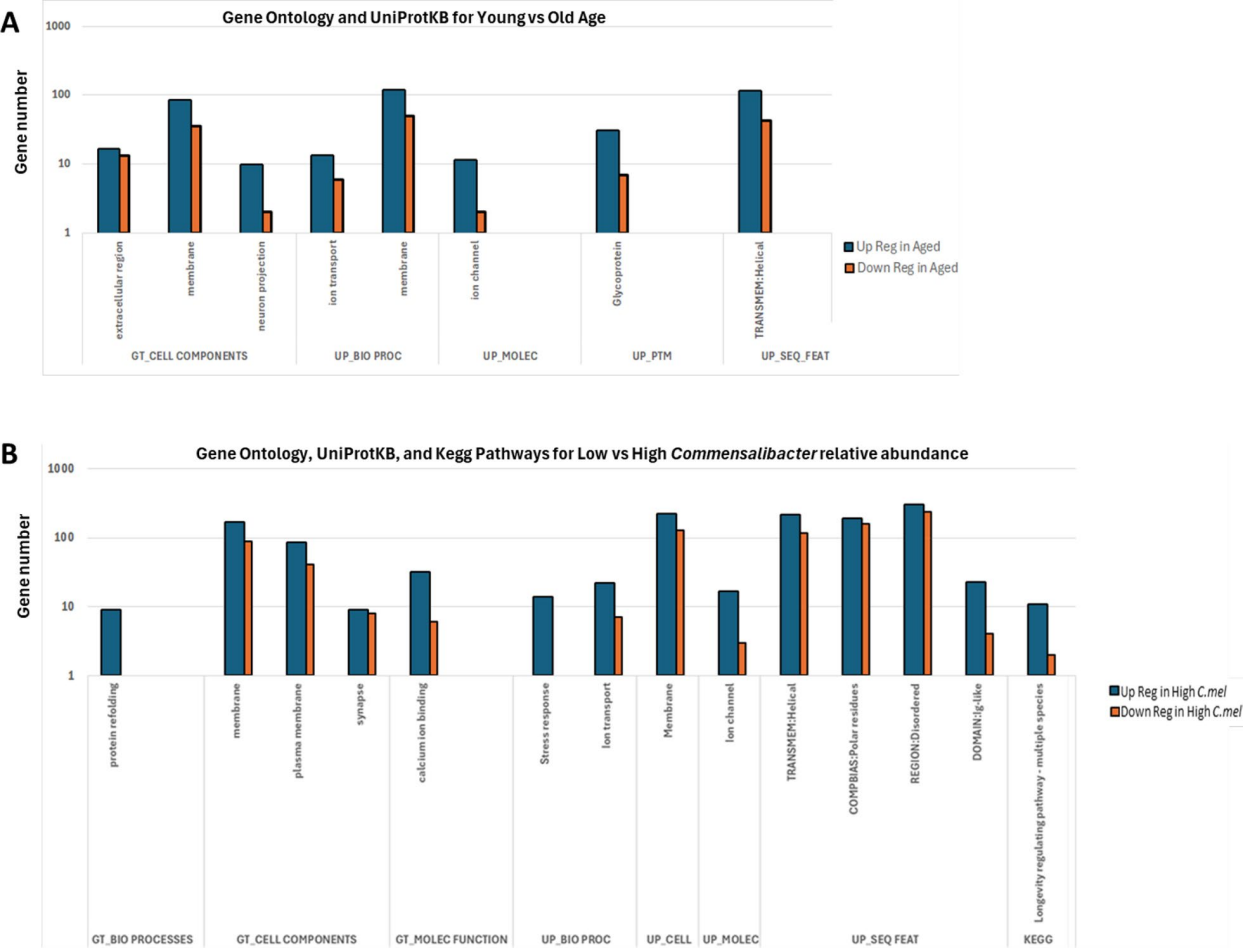
The counts of upregulated and downregulated genes for gene ontology (GO), UniProtKB (UP), and KEGG pathways for differentially expressed genes comparing age and *Commensalibacter melissae* relative abundance. Stacked bar charts show only significantly enriched annotation categories with a *p* *value* < 0.05 after FDR correction. **A** Functional annotations of differentially expressed genes for young and old queens. **B** Functional annotations of differentially expressed genes for queens with low and high *C. melissae* relative abundance. Abbreviations: GT, Go Term; BIO PROC, Biological Process; MOLEC, Molecular Function; UP BIO PROC, UniProtKB Key Words Biological Process; UP CELL, UniProtKB Cellular Component; UP MOLEC, UniProtKB Molecular Function; UP PTM, UniProtKB Post-translational modification; UP_SEQ_FEAT, UniProtKB sequence features

Functional annotation was performed on lists of DEGs for age and *C. melissae* relative abundance, including their shared and unique gene lists. In the comparison of young versus old queens, for GO terms, only cellular components had significant enrichment items; extracellular region, membrane, and neuron protection (Fig. 3A). There were fewer significantly enriched UP KW for biological process (2: ion transport and membrane), molecular function (1: ion channel), post-translational modification (1: glycoprotein), and UP sequence features (1: TRANSMEM:Helical). The functional annotation of the number of DEGs resolved 164 genes under the GO term cellular components, 194 for the UP KW biological process, 14 for UP KW molecular function, 39 for UP KW post-translational modification (PTM), and 162 for the UP KW sequence features (Table S10). The distribution of upregulated and downregulated genes (comparing youthful to aged) across different functional categories is visualized in Fig. 3A below.

There were more significant enrichment items associated with high versus low *C. melissae* relative abundance compared to the age-based analysis (Fig. 3B). For GO terms, protein refolding was enriched for biological process. Membrane, plasma membrane, and synapse for cellular components and calcium ion binding for molecular function. UP KW for biological process (2: stress response and ion transport), cellular component (1: membrane), molecular function (1: ion channel), and UP sequence features (4: transmembrane region of proteins that form helical structures, proteins that have a compositional bias towards polar amino acid residues, protein that lacks a fixed or ordered three-dimensional structure, and protein domains with an immunoglobulin-like fold). Additionally, *C. melissae* abundance was associated with significantly enriched KEGG pathway related to evolutionarily conserved longevity-regulating mechanisms identified across multiple model organisms. The functional annotation of the number of DEGs resolved 9 genes under the GO term biological process, 406 for cellular components, and 38 under molecular function. For UP KWs, we resolved 43 genes for biological process, 353 for cellular components, 20 for molecular function, and 1257 for sequence features (Table S11). The KEGG pathway for longevity had 13 genes enriched.

Given the relationship between age and *C. melissae* relative abundance, we used a Venn diagram analysis to distinguish genes uniquely associated with each factor and those shared between them (Fig. 2D). The 467 DEGs unique to age resolved one KEGG pathway under metabolic pathways for 37 genes (Table S12). The 984 DEGS unique to *C. melissae* showed significant enrichment for the GO term response to heat under biological process (7 genes) (Table S13). Notably, the KEGG pathway for longevity, including the 13 genes remained associated with *C. melissae* abundance. The 252 DEGs that were shared between age and *C. melissae* showed functional annotation aligned with GO term membrane for cellular component (83 genes) and UP KW cellular component (118 genes) (Table S14). UP KW biological process involving ion transport (14 genes) and UP KW molecular function ion channel (11 genes) were also enriched. UP KW PTM was enriched for glycoprotein (31 genes). Lastly, UP sequence features were enriched for transmembrane region of proteins that form helical structures (112 genes).

In summary, the functional annotation analysis revealed age-related DEGs were primarily associated with extracellular and membrane components, while *C. melissae* abundance-related DEGs showed a broader functional enrichment, including stress response, protein refolding, and longevity-related pathways. The KEGG pathway for longevity remained associated with *C. melissae* abundance even when analyzing unique DEGs not shared with age effects.

### qRT-PCR validation of differentially expressed genes in *C. melissae* and *A. mellifera*

**Fig. 4:**
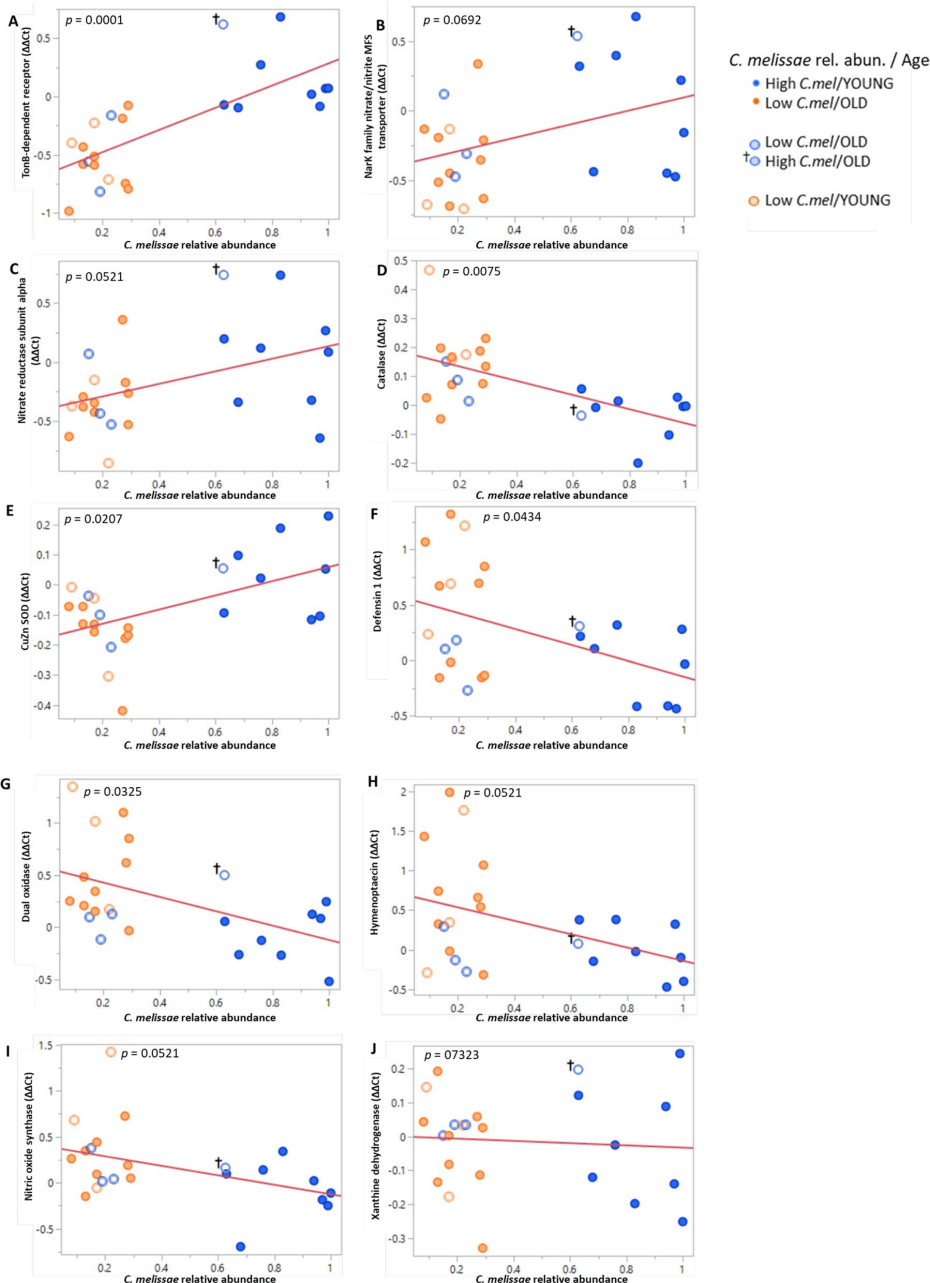
Regression analysis of gene expression versus *C. melissae* relative abundance. Each panel shows the relationship between gene expression (y-axis) and *C. melissae* relative abundance (x-axis) for selected genes from *C. melissae* (**A**–**C**) and *A. mellifera* (**D**–**J**). Expression values are normalized to β-actin and RPS5 reference genes using the 2 − ΔΔCT method and log-transformed. Points are colored according to the scheme in Fig. 2C showing youthful and aged categories with their respective outlier queens. Red lines show linear regression fits with corresponding *p* values shown in each panel. **A** TonB-dependent receptor, **B** NarK family nitrate/nitrite MFS transporter, **C** Nitrate reductase subunit alpha, **D** Catalase, **E** Cu–Zn superoxide dismutase, **F** Defensin 1, **G** Dual oxidase, **H** Hymenoptaecin, **I** Nitric oxide synthase, **J** Xanthine dehydrogenase

To further investigate the relationship between gene expression and *C. melissae* relative abundance, we performed linear regression analysis on RT-qPCR results from selected genes. For *C. melissae* genes, TonB-dependent receptor (*p* = 0.0001), NarK family nitrate/nitrite MFS transporter (*p* = 0.0692), and Nitrate reductase subunit alpha (*p* = 0.0521) all showed positive correlations with *C. melissae* relative abundance (Fig. 4A–C), indicating higher expression of these genes in queens with greater *C. melissae* abundance. These genes are involved in nutrient acquisition and nitrogen metabolism [51, 53], suggesting differences in bacterial metabolic activity across the gradient of *C. melissae* abundance.

For *A. mellifera* gene expression we observed significant relationships with *C. melissae* abundance for several genes with established roles in oxidative stress response and aging processes. Catalase and Cu–Zn superoxide dismutase, both critical antioxidant enzymes that neutralize reactive oxygen species [34], showed contrasting patterns: catalase showed a negative correlation with *C. melissae* abundance (*p* = 0.0075), with higher expression in queens with lower *C. melissae* abundance (Fig. 4D), while Cu–Zn superoxide dismutase exhibited a positive correlation with *C. melissae* abundance (*p* = 0.0207; Fig. 4E). The antimicrobial peptides defensin 1 (*p* = 0.0434) and hymenoptaecin (*p* = 0.0521), involved in immune function which often changes with age [21], as well as dual oxidase (*p* = 0.0325) and nitric oxide synthase (*p* = 0.0521), which generate reactive oxygen species and play roles in both immunity and aging, all showed negative correlations with *C. melissae* abundance (Fig. 4F–I). Xanthine dehydrogenase, involved in purine metabolism and reactive oxygen species generation, showed no significant relationship with *C. melissae* abundance (*p* = 0.7323; Fig. 4J), despite significance as a DEG. These patterns suggest that queens with higher *C. melissae* abundance tend to express lower levels of genes involved in antimicrobial defense and certain aspects of oxidative stress response, potentially reflecting differences in physiological state associated with variation in gut microbiome composition.

## Discussion

There are many examples of gut symbionts providing beneficial host effects including longevity [14, 17, 38]. In this study, we highlight contributions to longevity of the honey bee queen gut symbiont *Commensalibacter melissae* by combining 16S rRNA gene sequencing and transcriptomics. Our findings suggest that *C. melissae* relative abundance in the hindgut of honey bee queens is associated with significant changes in gene expression relative to age alone, with specific transcriptional patterns reflecting known models of longevity.

The microbial community analysis showed *C. melissae* (Alpha 2.1) and *Lactobacillus panisapium* (Firm-5) dominated the queen microbiome, comprising approximately 73% of the total hindgut microbiota. This is consistent with previous studies demonstrating these two groups/species as major contributors to the native queen gut microbiome [3, 42, 66]. Interestingly, while the relative abundance of *C. melissae* differed significantly between young and old queens, the absolute abundance did not, suggesting that the proportion of *C. melissae* in the gut community, rather than its absolute numbers, may be more relevant to queen health. In general, young queens had more of their microbiome dedicated to *C. melissae* than older queens (Fig. 1), confirming previous findings that *C. melissae* relative abundance depletes with age [3]. The mechanism for why queens lose *C. melissae* and its implications are still currently unknown, but may be driven by age-related changes to queen physiology and the gut environment. *C. melissae* belongs to the group of acetic acid bacteria (AAB) that form symbioses with insects, which typically thrive in acidic pH and with access to diet-derived carbohydrates [25]. Alterations in pH could result in a less hospitable environment to *C. melissae* over time, instead, supporting the growth of other symbionts like *Lactobacillus* and *Bifidobacterium* which increase with age [3]. Another explanation for the depletion of *C. melissae* with age could be a change in queen immune function, perhaps resulting in a queen becoming less tolerant of *C. melissae*. The immune system undergoes senescence with many changes throughout an organism’s lifespan which is reflected in youthful versus aged gene expression (Fig. 2).

### *Commensalibacter melissae* relative abundance and RNA-seq

Transcriptomic analysis uncovered substantial differences in gene expression patterns between young and old queens, as well as between queens with high and low *C. melissae* abundance. Our study confirmed that young queens typically have higher *C. melissae* relative abundance, however, gene expression patterns did not show complete correspondence with either age or bacterial abundance. The principal component analyses (Fig. 2) display distinct clustering of gene expression profiles based on both age and *C. melissae* abundance. Notably, the gene expression patterns associated with *C. melissae* abundance showed greater separation than those associated with age alone, suggesting that the gut symbiont abundance may have a more pronounced effect on queen physiology than age itself. In the analysis of age (Fig. 2B), we identified one high-performing queen with youthful gene expression signatures despite being more advanced in age. The analysis of *C. melissae* revealed three queens whose gene expression profiles diverged from expectations based on their age and bacterial abundance (Fig. 2C). Young queens with low *C. melissae* abundance exhibited gene expression patterns similar to older queens, suggesting an association between reduced symbiont abundance and age-related gene expression profiles, although the causal relationship remains to be determined. We found a similar result for queens relocated to a queen bank; an environment of low metabolic demand [20]. Our observations help explain why some young queens show reduced performance despite their age. Studies with *Drosophila* provide interesting parallels to our observations in honey bee queens. Similar to honey bees, the microbiota of fruit flies change in abundance and composition throughout aging [3, 12, 17]. Like honey bee queens, *Drosophila* aging research has revealed intriguing relationships with native Acetobacteraceae, including species of *Gluconobacter*, *Acetobacter*, and *Lactobacillus* [4]. However, a critical unknown in both systems is how bacterial products interact with host tissues. The physical proximity between bacterial populations and host cells necessary for metabolite signaling remains poorly understood. Thus, more research regarding the metabolites and host-microbe interactions of *C. melissae* and *A. mellifera* are needed.

Even among its insect-adapted sister genera (or 'congeners'), *C.melissae* appears uniquely derived and specialized, possessing both a reduced genome (having lost several pathways) and an excess of species-specific gene clusters [10]. In observation of aligned *C. melissae* genes, there were 9 DEGs shared between queens with low and high *C. melissae* abundance (Table S9), which may influence queen longevity through various mechanisms. For example, the differential expression of the TonB-dependent receptor gene suggests changes to microbial nutrient acquisition capabilities [53]. Efficient nutrient uptake and metabolism are crucial for maintaining cellular health and function over time. The upregulation of the TonB-dependent receptor in queens with high *C. melissae* abundance might mean they perform more efficient nutrient utilization, specifically the uptake of various siderophores, vitamins, and carbohydrates. The differential expression of the BadF/BadG/BcrA/BcrD ATPase family protein gene, which is involved in metabolite transport [28], suggests that *C. melissae* may modulate the metabolic environment of the queen's gut by facilitating the import or export of specific metabolites. Aldo/keto reductases are involved in various metabolic processes and stress responses, including the detoxification of reactive aldehydes and ketones that can accumulate with age [7]. Upregulation of this gene in queens with high *C. melissae* abundance could enhance stress resistance and detoxification capabilities for the host. There were several DEGs involved in ribosome function (16S rRNA genes), RNA processing (Ribonuclease J), and gene regulation (helix-turn-helix transcriptional regulator), which suggests that *C. melissae* may influence fundamental cellular processes. Maintaining efficient protein synthesis and gene regulation is important for cellular homeostasis and could contribute to slowing the aging process.

The elevated expression of NarK family nitrate/nitrite MFS transporter and nitrate reductase subunit alpha in youthful queens with high *C. melissae* abundance suggests a potential role in nitrogen recycling within the queen hindgut. NarK transporters are crucial for nitrate/nitrite transport across bacterial membranes [51], while nitrate reductase catalyzes the reduction of nitrate to nitrite [35], a key step in nitrogen metabolism. While it may be tempting to draw parallels with nutritional symbioses, several critical considerations challenge this interpretation. Unlike intracellular symbionts such as *Blattabacterium* in cockroaches or *Buchnera* in aphids, *Commensalibacter* sp. resides in the hindgut, physically separated from sites of nutrient absorption in the midgut. Without evidence of specialized transport mechanisms or gut chambers, the simplest explanation is that these nitrogen-metabolizing genes support bacterial metabolism rather than host nutrition. A more parsimonious hypothesis is that *C. melissae* uses host nitrogenous waste products as metabolic substrates, potentially influencing the succession of other gut microbes such as lactic acid bacteria. The decreased expression of these nitrogen-metabolizing genes in aged queens with lower *C. melissae* abundance might reflect changes in bacterial metabolism that accompany microbial succession in the aging gut. This is further supported by our finding that total bacterial abundance remains stable between young and old queens, despite the significant shift in community composition. This suggests that as queens age, the ecological niche occupied by *C. melissae* may be filled by other bacterial taxa, potentially altering the nitrogen metabolism dynamics in the hindgut. The constant overall bacterial population, coupled with changing relative abundances, indicates that microbial succession rather than overall bacterial depletion characterizes the aging queen gut. These patterns suggest that *C. melissae* may be particularly well-adapted to the gut environment of younger queens, with its metabolic activities potentially contributing to their physiological state.

The observations of two older queens with low *C. melissae* abundance and one old queen with high *C. melissae* abundance all displaying youthful gene expression patterns suggest complex interactions between multiple factors affecting queen quality. While our study focused on gut symbionts, queen quality and longevity are known to be influenced by other aspects including nutrition, mating success, colony conditions, seasonal effects, and environmental stressors. These high-performing older queens demonstrate that the relationship between *Commensalibacter* sp. and queen physiology is not strictly linear or deterministic. While there is a clear association between *C. melissae* abundance and youthful gene expression, the exceptions to this pattern indicate that queen quality is modulated by multiple interacting factors. These likely include colony-level factors such as worker population and resource availability, environmental conditions such as temperature and seasonal variation, and other members of the gut microbiome. This complexity aligns with previous research showing that queen quality cannot be reduced reliably to a single factor but rather emerges from the interaction of multiple biological and environmental variables.

### *Commensalibacter melissae* relative abundance and *Apis mellifera* differentially expressed genes

Functional annotation of *Apis mellifera* DEGs provide further insights into the biological processes affected by aging and *C. melissae*. In the age-associated DEGs, we observed enrichment of genes involved in the extracellular region, membrane, and neuron protection (Fig. 3C). The number of DEGs associated with *C. melissae* abundance doubled those of age, and showed enrichment across a broader range of categories and functions, including protein refolding, stress response, ion transport, and calcium ion binding. The enrichment of stress response genes is interesting, as it implies that *C. melissae* may influence queens'stress response mechanisms. Notably, there were 13 DEGs significantly enriched for the KEGG pathway in longevity-regulating pathways, suggesting that *C. melissae* relative abundance may influence queen longevity through modulation of these genes. Specifically, genes that encompass a range of functions critical to cellular stress response, metabolism, and longevity regulation. Among these genes, catalase (Cat) and superoxide dismutase [Cu–Zn] (SOD) are noteworthy as they play crucial roles in the antioxidant defense system. These enzymes are responsible for neutralizing reactive oxygen species (ROS), which are implicated in cellular damage and aging [34, 37]. Catalase converts the ROS hydrogen peroxide to water and oxygen and was lower in youthful queens with high *C. melissae* abundance suggesting lower levels of oxidative stress. Conversely, Cu–Zn SOD, which catalyzes the dismutation of extremely unstable superoxide radicals to hydrogen peroxide and molecular oxygen [46], was highest in youthful queens. The elevated expression of Cu–Zn SOD in queens with high *C. melissae* abundance likely reflects higher levels of intracellular mitochondrial activity—as increased energy production generates more ROS, necessitating increased Cu–Zn SOD expression. The lower catalase expression in these queens suggests that despite higher metabolic activity, they may be managing ROS more efficiently at the superoxide stage. This pattern indicates that *C. melissae* abundance correlates with queens maintaining high metabolic activity while effectively managing oxidative stress.

The heat shock proteins (Hsp70 Ab-like, Hsc70-4, and multiple lethal (2) essential for life variants) identified in the KEGG pathway analysis of *A. mellifera* DEGs are crucial for protein folding and cellular stress responses [68]. The induction of heat-shock proteins, particularly Hsp70, has been associated with aging and proteostatic stress across various model systems [43] as well as with honey bees [63]. Transcripts were highest in queens with low *C. melissae* abundance, suggesting that they may be experiencing higher levels of proteostatic stress. The increased expression of heat shock proteins could represent a compensatory mechanism attempting to maintain protein homeostasis in the face of age-related cellular stress or the absence of protective effects modulated by the presence of *C. melissae*. Furthermore, the presence of multiple lethal (2) essential for life variants among the differentially expressed genes is particularly intriguing. These genes, which are homologs of the human HSPA1 A (Heat Shock 70 kDa Protein 1A), are crucial for cellular survival under stress conditions [64]. Their lower expression in queens with high *C. melissae* abundance could indicate a reduced need for these stress-response mechanisms, possibly due to a more stable cellular environment promoted by the presence of the symbiont. These findings align with the concept of hormesis, where mild stress can lead to improved stress resistance and longevity [55]. It's possible that *C. melissae* provides a low level of beneficial stress or stimulates protective pathways in the host, leading to improved stress resistance without the need for constant high expression of stress response genes. This could result in a more efficient use of cellular resources and potentially contribute to the exceptional longevity of honey bee queens.

### Study limitations

Several important limitations of this study should be acknowledged. First, these findings are correlational in nature, and we cannot establish causal relationship between *C. melissae* abundance and queen physiology or gene expression patterns. Future research should attempt experimental manipulations of the queen microbiome through feeding monocultures and/or creating germ-free queens. Second, our focus on *C. melissae* raises the question of whether this specific symbiont is uniquely influential, or if other community members that change with age might play equally important roles. While our data show that *C. melissae* relative abundance correlates most strongly with the gene expression changes we identified, the reciprocal increase in *Lactobacillus* and other taxa as *C. melissae* decreases could also influence host physiology. Disentangling these possibilities would require selective removal and reintroduction of specific microbial community members. Third, our sampling approach captured a single timepoint in a queen’s lifespan, thus limiting our ability to track true longitudinal changes in microbiome composition and gene expression. Fourth, while carbonyl accumulation serves as a useful biomarker for biological age, it represents only one aspect of the complex aging process. Finally, the use of categorical comparisons, while valuable for identifying distinct gene expression profiles, may not fully capture the continuous and potentially non-linear relationships between microbiome composition, age, and gene expression. Future work must employ a combination of experimental manipulations, longitudinal sampling, and additional aging biomarkers to tease apart the complex relationships between the gut microbiome, queen longevity, and quality.

## Conclusion

The findings from this study provide novel insights into the role of the honey bee queen gut symbiont, *Commensalibacter melissae* (*C. melissae*), in modulating queen physiology. Our results suggest that the relative abundance of *C. melissae* in the queen hindgut is intimately associated with distinct gene expression patterns, which diverge more strongly based on symbiont abundance than age alone. Younger queens typically harbor higher relative abundances of *C. melissae* compared to older queens. While this suggests a potential relationship between *C. melissae* abundance and aging processes, further experimental studies are needed to determine whether changes in microbial composition are a cause or consequence of aging in queens. The occurrence of gene expression profiles that do not align with chronological age suggests a complex, non-linear relationship of the queen hindgut microbiome with host age. In conclusion, the queen aging appears to be intimately linked to the presence and relative abundance of the gut symbiont *C. melissae*. Further investigation into the specific mechanisms underlying this relationship could provide valuable insights into the regulation of aging in social insects.

## Supplementary Information

Additional file 1

## Data Availability

The dataset generated for this study can be found in the GenBank, Sequence Read Archive as BioProject PRJNA1207053. The RNA-Seq data were deposited in the Gene Expression Omnibus (GEO) of the National Center for Biotechnology Information and can be accessed with the GEO accession number GSE GSE286382 (https://www.ncbi.nlm.nih.gov/geo/query/acc.cgi?acc=GSE286382).
